# Chloroform in Various Diseases

**Published:** 1849-04

**Authors:** 


*Chloroform in various diseases. In Convulsions.*—Professor Jackson referred to the history of a case remarkable for the quantity of chloroform that was used. A lady laboring under a stricture of the upper portion of the rectum which prevented the flatus from passing, became, in consequence, the subject of an enormous distension of the abdomen, attended with so great a degree of sensitiveness that the use of palpitation and percussion were entirely precluded, and her case was at first involved in no little obscurity.

In the latter part of the month of December, 1845, she was attacked by a violent convulsive paroxysm, preceded by a very peculiar spasmodic affection, consisting in a cracking of the head of the humerus in the glenoid cavity and of the femur in the acetabulum; these spasmodic symptoms continued for about fifteen minutes, when general convulsions set in, accompanied with intense pain. On the 1st of January, 1846, an attack of spasms occurred and continued for several hours; the urine was retained from a spasmodic affection of the neck of the bladder and urethra, the orifice of which latter was so much retracted that it was difficult to introduce a catheter to relieve the distension of the bladder. A few drops of chloroform were given to the patient to inhale, and prompt relief was experienced. The use of the chloroform was continued daily for two or three weeks, the quantity being gradually increased as the effects diminished. Dr. J. was sent for one morning, and found the mother of the lady in great alarm in consequence of the quantity of chloroform which her daughter had taken. She had inhaled two ounces in the course of the evening, then two ounces more, and an additional ounce in the course of the night; being five ounces inhaled from 5 o'clock, P.M., until 10 o'clock of the ensuing morning. Dr. J. found her with feeble pulse, diminished temperature of the body, and considerable excitement of mind. She insisted upon having more of the ether to inhale. She remained cold and nearly pulseless for forty-eight hours; when all the effects of the inhalation disappeared, and, what is remarkable, since that time she has had no return of her spasms. Upon one occasion having a tooth taken out, the pain of the operation caused a tendency to their return, but this went off without the spasms occurring.

*In Trismus Nascentium.*—Dr. BOND related a case of trismus nascentium which occurred in the infant of a female who had been delivered after an easy labor without any medical attendant. When Dr. B. was called to the infant, it was a week old, and exhibited the symptoms of trismus well-marked. He administered a mixture of chloroform and assafetida internally, and the child recovered. How far this result was to be attributed to the chloroform, Dr. B. is not prepared to say, inasmuch as other remedies were employed at the same time. The bowels of the infant were much disordered and soon as they were restored by an appropriate treatment to a healthy condition, the trismus began to disappear.

*In Neuralgia.*—Dr. HAYS stated, that he had employed chloroform to produce local anæsthesia with apparently the most happy effects, in a case of neuralgia, occurring in a gentleman 50 years of age, who had been for a long time a sufferer from neuralgia of the foot, in which all the remedies that had been previously employed failed to produce relief. Dr. H. was called to this patient about eighteen days since, and found him in intense pain, which had deprived him of sleep the whole of the preceding night. Dr. H. directed the affected parts to be enveloped with a pledgit of lint or a few folds of muslin wet with chloroform, and the whole to be covered with a portion of oiled silk to prevent evaporation; on the next morning he found him entirely free from pain, which has not since returned. Whether the relief experienced in this case is to be ascribed to the local anæsthesia produced by the chloroform, or is to be considered as a mere coincidence, Dr. H. does not pretend to decide.

*In Labor.*—Dr. PARRISH stated, that a case of labor in which chloroform was used, had been communicated to him by his brother, Dr. Joseph Parrish, of Burlington, N. J. He was called a few days since to attend a case of protracted labor from rigidity of the os uteri, attended with severe ineffectual pains; he employed a mixture of ether and chloroform; almost immediately the parts became relaxed, and the labor proceeded with increased activity. The child was soon born, and without any consciousness on the part of the mother.

Dr. GRISCOM remarked, that he had employed ether inhalation, as remarked in the report read by him this evening, in two cases of labor. The first was a protracted, instrumental, case; the female had been in labor twenty-four hours. There was great rigidity of the parts and dryness of the vagina. The patient was very intolerant of pain. As soon as she commenced inhaling the ether she pronounced herself as in heaven. There quickly took place a copious secretion of the vaginal mucus, and the child was soon delivered. The other case was one of natural labor, but attended with severe pains. The ether was administered at the patient's desire. She did not inhale a sufficient quantity to produce unconsciousness, but acknowledged very decided relief from its use.

*In Nephritic Colic.*—Dr. STILLE stated, that last spring Dr. Bowditch, of Boston, related to him a case of nephritic cholic, occurring in an individual who had previously suffered from several attacks, in which the chloroform had been administered with the effect of inducing entire relief of the pain, without abolishing consciousness. The influence of the chloroform was kept up for several hours, when, at length, the stone passed through the ureter. Within the last three weeks a lady, about

20 years of age, after retiring to bed some hours, was attacked with violent pain in the region of the right kidney. About an hour afterwards (1 o'clock, A.M.,) Dr. Stillé was sent for. The mother had applied warm fomentations to the loins, and immersed the feet in a mustard bath. There was still intense pain, with tenderness over the region of the right kidney. The patient was lying bent double, her hands and feet were cold, her pulse feeble, and about sixty in the minute. The pain extended in the direction of the ureter; there was pain and a twitching motion of the right thigh, with frequent inclinations to urinate. From a review of the symptoms, Dr. S. considered the case to be one of nephritic cholic. The use of ether was suggested. A teaspoonful was poured upon a handkerchief and inhaled for five minutes, when the patient fell asleep. In about fifteen minutes she was aroused by a return of the pain. The ether was again inhaled, and its impression was kept up for about an hour, without carrying it so far as to abolish consciousness; the pain ceased, the extremities became warm, and the pulse rose to seventy-five. The next morning there was no pain complained of, but considerable soreness was experienced when pressure was made over the right kidney. The pain did not return until two nights afterwards, when a larger dose of ether was used, by the patient, without Dr. Stillé's presence or direction, but from some cause, it did not produce the same alleviation of the pain as before. At 4 o'clock in the morning Dr. S. saw her. The pain had by this time somewhat abated, and upon the administration of an opiate enema complete relief was procured. The pain has not since returned. No calculus was passed, so far at least, as could be ascertained.—*Summary Trans. Coll. Phys. Phila.*, Jan. 1849.

